# Amphiphilic Poly(dimethylsiloxane-ethylene-propylene oxide)-polyisocyanurate Cross-Linked Block Copolymers in a Membrane Gas Separation

**DOI:** 10.3390/membranes11020094

**Published:** 2021-01-29

**Authors:** Ilsiya M. Davletbaeva, Ilgiz M. Dzhabbarov, Askhat M. Gumerov, Ilnaz I. Zaripov, Ruslan S. Davletbaev, Artem A. Atlaskin, Tatyana S. Sazanova, Ilya V. Vorotyntsev

**Affiliations:** 1 Department of Synthetic Rubber, Kazan National Research Technological University, 68 Karl Marks str, 420015 Kazan, Russia; ilgiz-9393@bk.ru (I.M.D); gumerov_a@mail.ru (A.M.G.); 2 SIBUR LLC, 16, bld.3, Krzhizhanovskogo str., GSP-7, 117997 Moscow, Russia; zaripovilnaz@gmail.com; 3 Kazan National Research Technical University n.a. A.N. Tupolev—KAI, 10 Karl Marks str., 420111 Kazan, Republic of Tatarstan, Russia; darus@rambler.ru; 4 Laboratory of Membrane and Catalytic Processes, Nizhny Novgorod State Technical University n.a. R.E. Alekseev, 24 Minin str., 603950 Nizhny Novgorod, Russia; atlaskin@gmail.com (A.A.A.); yarymova.tatyana@yandex.ru (T.S.S.); ilyvootyntsev@gmail.com (I.V.V.); 5 Mendeleev University of Chemical Technology of Russia, Miusskaya Sq. 9, 125047 Moscow, Russia

**Keywords:** membrane gas separation, cross-linked block copolymers, core-shell, polyisocyanurates, carbon dioxide, methane, nitrogen

## Abstract

Amphiphilic poly(dimethylsiloxane-ethylene-propylene oxide)-polyisocyanurate cross-linked block copolymers based on triblock copolymers of propylene and ethylene oxides with terminal potassium-alcoholate groups (PPEG), octamethylcyclotetrasiloxane (D_4_) and 2,4-toluene diisocyanate (TDI) were synthesized and investigated. In the first stage of the polymerization process, a multiblock copolymer (MBC) was previously synthesized by polyaddition of D_4_ to PPEG. The usage of the amphiphilic branched silica derivatives associated with oligomeric medium (ASiP) leads to the structuring of block copolymers via the transetherification reaction of the terminal silanol groups of MBC with ASiP. The molar ratio of PPEG, D_4_, and TDI, where the polymer chains are packed in the “core-shell” supramolecular structure with microphase separation of the polyoxyethylene, polyoxypropylene and polydimethylsiloxane segments as the shell, was established. Polyisocyanurates build the “core” of the described macromolecular structure. The obtained polymers were studied as membrane materials for the separation of gas mixtures CO_2_/CH_4_ and CO_2_/N_2_. It was found that obtained polymers are promising as highly selective and productive membrane materials for the separation of gas mixtures containing CO_2_, CH_4_ and N_2_.

## 1. Introduction

Block copolymers, including multiblock copolymers of an amphiphilic nature, are currently attracting research efforts due to their ability to form various supramolecular structures [1,2,3,4,5,6,7,8,9,10,11,12,13,14,15,16]. The direct influence on the supramolecular organization of block copolymers is a way of controlling both the mechanical and physicochemical properties of polymer materials obtained on this basis.

Polyorganosiloxane block copolymers have been widely investigated [17,18,19,20,21,22,23,24,25,26,27,28]. Such copolymers combine the specific practically useful properties of polydimethylsiloxanes (hydrophobicity, high gas permeability and low free surface energy) with the hydrophilicity, heat sensitivity, crystallization ability and relatively low melting point of poly(ethylene oxide) blocks. Polyorganosiloxane block copolymers have surface-active properties; therefore, they are used as emulsifiers and emulsion stabilizers.

The unique properties of polysiloxanes (hydrophobicity, gas permeability, low glass transition temperature, low surface energy) with hydrophilicity, heat sensitivity, bio- and hemocompatibility of oligoethyleneoxide are manifested in siloxane(ethylene oxide)urethane block copolymers [29,30]. The absence of Si–O–C bonds in their structure ensures hydrolytic stability, and the presence of urethane groups which can form hydrogen bonds have an additional effect on the formation of various structures in the polymer matrix.

Membranes based on polyorganosiloxane block copolymers can exhibit high permeability for various gases and good mechanical strength [31,32,33,34,35,36,37,38,39]. Basically, polyorganosiloxane block copolymers are heterogeneous systems. The degree of microphase separation can vary according to the origin of the blocks in the copolymer, their length and the molecular weight distribution of the fragments along the chain. As a result, materials with significantly different properties can be synthesized. If the phase separation is good, the main parameters of the membrane material (permeability, selectivity, mechanical properties) are determined by the fragment that prevails in this material and forms a continuous phase. At high polydimethylsiloxane phase content, the domains of the hard blocks are spherical. With a decrease in the degree of phase separation, the contribution of both copolymer blocks affects these properties. Therefore, it is extremely important to know the limits of phase segregation to solve specific problems of membrane separation. The morphology, sizes, and shapes of the structures (domains) of various blocks in polyorganosiloxane block copolymers strongly influence the diffusion properties of heterogeneous systems.

The wide possibilities for controlling both the macromolecular and supramolecular structures are associated with multiblock copolymers obtained on the basis of terminated by potassium–alcoholate groups of triblock copolymers of propylene and ethylene oxides (PPEG) and 2,4-toluene diisocyanate (TDI) [15,37,38,39]. The high activity of the potassium-alcoholate groups of PPEG in the reactions of the opening of isocyanate groups initiated by them by the anionic mechanism was established. Depending on the created reaction conditions, the interaction of PPEG with TDI can be accompanied by the formation of both coplanar acetal-type polyisocyanate blocks and branched polyisocyanurate structures.

In the works [40,41,42], copolymers based on PPEG, octamethylcyclotetrasiloxane (D_4_), and TDI were obtained. The peculiarity of the copolymerization consisted in the opening of isocyanate groups occurred via carbonyl component activated by potassium–alcoholate groups of PPEG and the subsequent polymerization of octamethylcyclotetrasiloxane initiated by the oxanion. It was found that Si–O–C bonds, being an integral part of the resulting polymer matrix, are not subjected to hydrolytic effects. Preliminary studies have shown that this approach is promising for a directed influence on the supramolecular organization and the design of polymeric gas separation membranes [43,44,45,46].

**Scheme 1:**
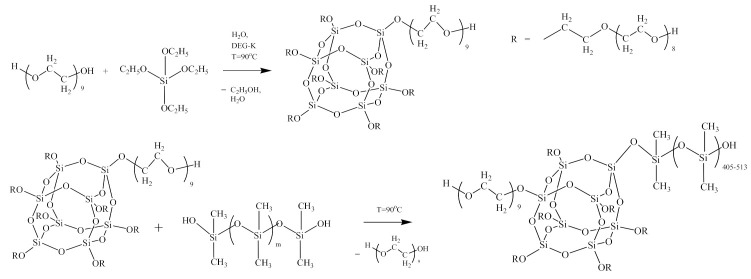
Amphiphilic branched silica derivatives associated with oligomeric medium (ASiP) synthesis scheme.

Amphiphilic silica derivatives (ASiP) were synthesized in the work [47] (Scheme 1). It was found that ASiP exhibit co-catalytic activity in the D_4_ polyaddition reactions by the anionic mechanism and the ability to be located at the boundary of thermodynamically incompatible phases and, thus, affect the relative position of the components of multi-block copolymers during their formation.

The main objective of this study is the synthesis of amphiphilic poly(dimethylsiloxane-ethylene-propylene oxide)-polyisocyanurate cross-linked block copolymers in the presence of ASiP and the study of the features of their supramolecular structure and gas transport properties.

## 2. Materials and Methods

### 2.1. Materials

The block copolymer of propylene and ethylene oxide (PPEG) with formula HO[CH_2_CH_2_O]_n_[CH_2_(CH_3_)CH_2_O]_m_[CH_2_CH_2_O]_n_K, where n ≈ 14 and m ≈ 48, molecular weight 4200 g/mol, containing 30 wt.% of peripheral polyoxyethylene blocks, where the content of potassium alcoholate groups is 10.9% from the total number of functional groups, was purchased from PJSC Nizhnekamskneftekhim (Nizhnekamsk, Russia). Octamethylcyclotetrasiloxane (D_4_) was purchased from Nanjing Union Silicon Chemical Co., Ltd. (USI Chemical, Nanjing, China). Amphiphilic branched silica derivatives associated with oligomeric medium (ASiP) was obtained in laboratory conditions [40]. 2,4-toluene diisocyanate 98 % (TDI) was purchased from Sigma-Aldrich (St. Louis, MO, USA). PPEG was additionally dried at reduced pressure (approximately 0.1 kPa) and at an elevated temperature of 95 °C down to 0.01% moisture concentration.

In the gas separation investigation of prepared polymers, gases (methane, carbon dioxide, nitrogen) were used with purity no less than 99.995% (NII KM, Moscow, Russia).

### 2.2. Synthesis of Polymers Based on PPEG, D_4_, TDI

The reaction was carried out in two stages. In the first stage, multiblock copolymer (MBC) was obtained by the interaction of PPEG with D_4_ in their molar relations [D_4_]:[PPEG] = 2, 5, 7, 8, 9, 10, 15. ASiP were used as a modifier. The calculated amount of PPEG and D_4_, as well as ASiP as a modifier, was placed in a round-bottom flask equipped with a reflux condenser, stirred for thirty minutes at T = 60 °C. Before conducting studies, MBC was preheated under a residual pressure of 0.7 kPa until they reached a constant weight.

In the second stage, polymers were obtained by the interaction of MBC with TDI in their molar ratios [PPEG]:[D_4_]:[TDI] = 1:2:12, 1:15:8, 1:15:15. Polymers were also prepared on the basis of PPEG and TDI in their molar ratios [PPEG]:[TDI] = 1:8, 1:12. The reaction was carried out in toluene at 70 °C in a flask equipped with a reflux condenser. The polymerization process was carried out with constant stirring using a magnetic stirrer. The reaction mass was stirred at this temperature until complete dissolution of the PPEG or MBC, and then introduced TDI. Five minutes after this, the reaction mass was poured into a Petri dish, and then it was cured at room temperature for 72 h.

### 2.3. Fourier Transform Infrared Spectroscopy Analysis (FTIR)

The FTIR spectra of the products were recorded on an InfraLUM FT 08 Fourier transform spectrometer (Lumex, St. Petersburg, Russia) using the attenuated total reflection technique. The spectral resolution was 2 cm^−1^, and the number of scans was 16.

### 2.4. Measurements of the Surface Tension

The droplet-counting method was used to determine the surface tension (σ). The basis of the calculations is the equation where the weight of the drop that comes off the pipette is proportional to the surface tension of the fluid and the radius of the pipette (R): m = 2π × R × σ/g, where g is acceleration of gravity; m is the drop mass of the test liquid. Following this equation: σ = mg/2πR. Further, according to the obtained results, a characteristic curve of the surface tension (σ) from concentration (C) was constructed.

### 2.5. Light-Scattering

Dynamic light-scattering experiments were carried out on Zetasizer Nano ZS (Malvern, Great Britain). This instrument has 4 mV He–Ne laser, which works on the 632.8 nm wavelength. Measurements were carried out at the 173° detection angle. The experiments were carried out at 25 °C in the disposable plastic cuvettes of 1 cm path length.

### 2.6. Measurement of Temperature Dependence of Dielectric Loss Tangent

The temperature dependence of the dielectric loss tangent (DLT) of polymer samples (thickness of 0.5–0.7 mm) was registered in the temperature range from −150 to 100 °C at a frequency of 1 kHz. A facility consisted of the measuring cell that was placed in a Dewar vessel filled with nitrogen, and to which an E7-20 RLC-meter and a B7-78 universal voltmeter functioning as a precision thermometer were connected.

### 2.7. Thermomechanical Analysis

The thermomechanical curves of polymer samples were obtained using TMA 402 F (Netzsch, Selb, Germany) thermomechanical analyzer in the compression mode. The sample thickness was 2 mm, and the rate of heating was 3 °C/min from −100 to 350 °C in the static mode. The load was 2 N.

### 2.8. Mechanical Loss Tangent Measurements (MLT)

The MLT curves of polymer samples were taken using the dynamic mechanical analyzer DMA242 (Netzsch, Selb, Germany) in the mode of the oscillating load. The force and stress–stain correspondence was calibrated using a standard mass. The thickness of the sample was 2 mm. Viscoelastic properties were measured under nitrogen. The samples were heated from −100 to 350 °C at the rate of 3 °C/min and a frequency of 1 Hz. The mechanical loss tangent was defined as the ratio of the viscosity modulus G” to the elasticity modulus G’.

### 2.9. Atomic Force Microscopy Analysis (AFM)

The obtained membrane surface was carried out by atomic force microscopy (AFM) also. Atomic force microscope SPM-9700 (Shimadzu, Kyoto, Japan) with a maximum scanner area equal to 30 μm was used. Because the testing material was a rather soft polymer, scanning was performed in the tapping mode with cantilever POINTPROBE FMR-20 silicon vibration (Nano World Innovative Technologies, Neuchatel, Switzerland) with a spring constant of 1.3 N m^−1^ and a typical tip curvature radius of definitely no more than 12 nm; the tip height varied from 10 to 15 μm. The cantilever with a curvature radius of tip no more than 8 nm was used for the observation of a topographic image to minimize the error introduced by the cantilever due to the narrowing of profile recesses. The AFM experiments were carried out at room temperature. The treatment of the resulting AFM images was realized with the aid of the SPM Manager^®^ ver. 4.02 software (Shimadzu, Kyoto, Japan). The samples with size 10% 15 mm were cleaned of dust, and then affixed to the center of the sample holder using a two-sided carbon tape (SPI Supplies Division of Structure probe Inc., West Chester, PA, USA).

### 2.10. Permeability Measurements

**Figure 1:**
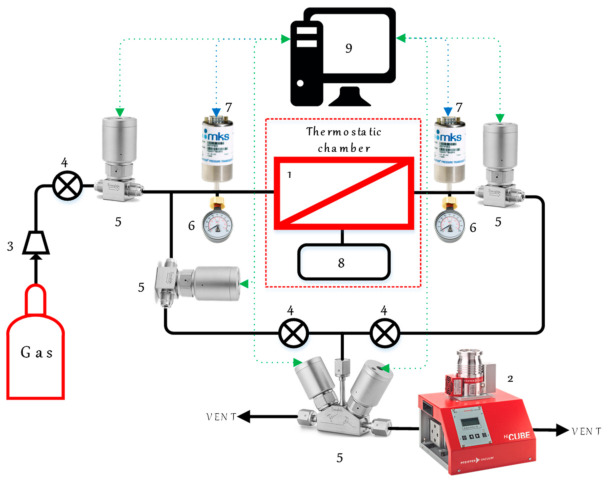
Schematic of experimental setup for single gas permeability test: 1—permeation cell; 2—vacuum station; 3—pressure reducer; 4—manual valves; 5—pneumatic valves; 6—pressure gauges; 7—pressure transducers; 8—additional volume for the permeate side; 9—workstation.

Gas permeability through the studied polymers was measured according to the well-known time-lag technique proposed by H.A. Dynes and R.M. Barrer [48,49], using a constant-volume variable-pressure apparatus [50,51,52] schematically shown in Figure 1. The setup is equipped with a typical permeation test cell made of AISI316 stainless steel with PTFE sealing, where the permeate side is connected to a pump station HiCube 80 Eco (Pfeiffer Vacuum, Aslar, Germany) providing vacuum up to 1 × 10^−5^ Pa. The active membrane area available for permeation is 5.3 cm^2^. The feed- and permeate-side pressures were monitored by high-precision pressure transducers: WIKA S-10 0–1.6 MPa (Wika, Klingenberg on Main, Germany) having an accuracy of 0.5% of span and MKS Baratron 750B 0–13.3 kPa (MKS Instruments, Andover, MA, USA) with an accuracy of 1% of reading. Initial feed pressure was maintained at 110 kPa. Each single-gas test was repeated at least three times at least 24 h apart before each run.

Permeability coefficient *P* was calculated according to

$$P=\frac{Vp_{2}T_{0}}{V_{m}P_{0}T}\frac{l}{S\tau \left(p_{1}-p_{2}\right)}$$

Moreover, the time-lag technique allows determine the diffusion coefficient (*D*). Furthermore, the sorption coefficient might also be easily calculated

$$D=\frac{l^{2}}{6\theta },\;S=\frac{P}{D}$$

where *V*—permeate-side volume; m^3^; *V_m_*—molar volume m^3^/mol; *p*_2_—permeate-side pressure, Pa; *p*_1_—feed-side pressure, Pa; *P*_0_—atmospheric pressure, Pa; *T*—temperature, K; *T*_0_—273.15, K; *S*—membrane area, m^2^; *l*—membrane thickness, m, *τ*—time of the experiment, *s*; *θ*—time lag, *s*. Thus, the permeability coefficient was found in the International System of Units of mol m m^−2^s^−1^Pa. The permeability coefficient is represented in Barrer unit (1 Barrer = 3.348 10^−16^ mol m m^−2^ s^−1^ Pa^−1^ = 10^−10^ cm^3^(STP) cm cm^−2^ s^−1^ cmHg^−1^). The thickness of studied samples was: 120, 155, 250 and 270 µm for [PPEG]:[TDI] = 1:10, [PPEG]:[D4]:[TDI] = 1:15:10, [PPEG]:[D4]:[TDI] = 1:15:10 [ASiP] = 0.2 wt.% and [PPEG]:[TDI] = 1:15, respectively.

The permeability coefficient relative error for a single test is less than 5%; consequently, the selectivity relative error is 7.1%. The relative error of the measurement series was usually less than 15%. Taking into account the thickness measurement relative error of 2.4%, the diffusion coefficient relative error is 5.5% and the sorption coefficient relative error equals 7.5%.

After permeability measurements, the ideal selectivity is calculated as the ratio of the fast over the slow permeability coefficients for the following pair of gases: CO_2_/CH_4_ and CO_2_/N_2_.

## 3. Results and Discussion

### 3.1. Synthesis and Physico-Chemical Characterization of MBC Based on PPEG and D_4_

The progress of the copolymerization of PPEG with D_4_ initiated by the terminal –O–K^−^ groups was investigated primarily by the homogenization of the reaction system. In the case of homopolymerization of D_4_, the resulting polydimethylsiloxane (PDMS) and PPEG are not mixed due to their thermodynamic incompatibility. As a result, the phase separation is clearly traced. During the course of copolymerization, the viscosity of the reaction system increases and a homogeneous opaque mass is formed. Before the studies, the copolymers were preheated under vacuum pressure until they reached a constant mass. D_4_ conversion was calculated gravimetrically, and in all cases, it exceeded 98 wt.%.

The possibility of copolymerization PPEG with D_4_ initiated by terminal potassium-alcoholate groups was also investigated by FTIR spectroscopy.

**Figure 2:**
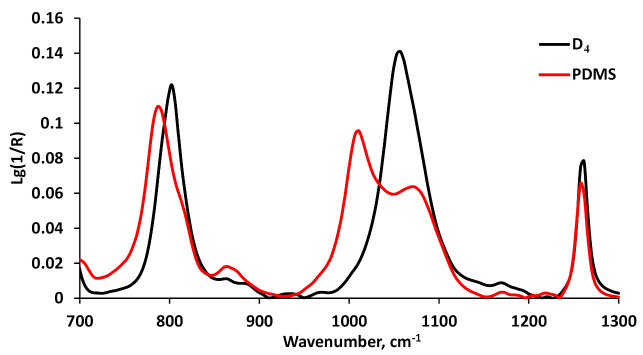
FTIR spectra of D_4_ and polydimethylsiloxane (PDMS).

For comparison, the FTIR spectra of D_4_ and polydimethylsiloxane are shown in Figure 2. An analytical band in the 787 cm^−1^ region, corresponding to the stretching vibrations of the Si–C bond and bands in the regions of 1011 and 1073 cm^−1^ due to the stretching vibrations of the Si–O–Si bond, is present in the FTIR spectrum of PDMS.

The D_4_ FTIR spectrum contains an analytical band at 802 cm^–1^, corresponding to the stretching vibrations of the Si–C bond and the band at 1057 cm^−1^, due to the stretching vibrations of the Si–O–Si bond.

**Figure 3:**
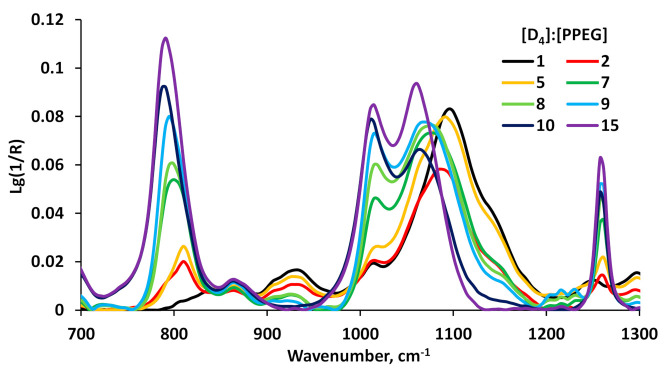
FTIR spectra of multiblock copolymer (MBC) obtained on the molar excess of D_4_.

The FTIR spectra of the products of the interaction of PPEG and D_4_ (Figure 3) at a low D_4_ content ([D_4_]:[PPEG] = 1 and 2) show the band at 810 cm^−1^, corresponding to the stretching vibrations of the Si–C bond as part of D_4_. With an increase in the mole fraction of D_4_ above [D_4_]:[PPEG] = 2, the band at 791 cm^−1^ is observed in the spectra, which corresponds to the stretching vibrations of the Si–C bond in PDMS.

The formation of polydimethylsiloxane chains as a result of the PPEG-initiated polyaddition of D_4_ is confirmed by FTIR spectroscopy—the appearance of the band at 791 cm^−1^ and its intensity increasing with increasing mole fraction of D_4_ in the reaction system. In addition, the bands at 1011 and 1076 cm^−1^ appear due to the stretching vibrations of the Si–O–Si bond in PDMS for the D_4_ polyaddition products to PPEG.

In the range of 1003–1096 cm^−1^, unusual changes are observed, which are manifested in the FTIR spectra of PPEG and D_4_ copolymers obtained with a relatively high molar excess of D_4_ disappearing at 1096 cm^−1^, which characterizes the C–O–C bonds in the composition of the used PPEG. With a relatively low molar excess of D_4_, on the contrary, only bands characterizing the C–O–C bonds in the composition of the used PPEG are found in the FTIR spectra.

To explain the observed phenomenon, it should be noted that the measurements were carried out in the mode of incomplete reflection. That is, infrared rays affected only the surface of the sample. Considering the amphiphilicity of the PPEG- and D_4_-based multiblock copolymers, it can be assumed that, due to the incompatibility of the hydrophilic polyether and hydrophobic polydimethylsiloxane components in the melt of the formed MBC, microphase separation processes occur. As a result, with an excess of D_4_, polydimethylsiloxane chains are predominantly located on the surface of the supramolecular formation (possibly a micellar structure). When D_4_ is deficient, on the contrary, the micelle surface consists of polyether chains. Thus, the conducted FTIR spectroscopic studies confirm the formation of MBC during the interaction of PPEG with D_4_.

**Figure 4:**
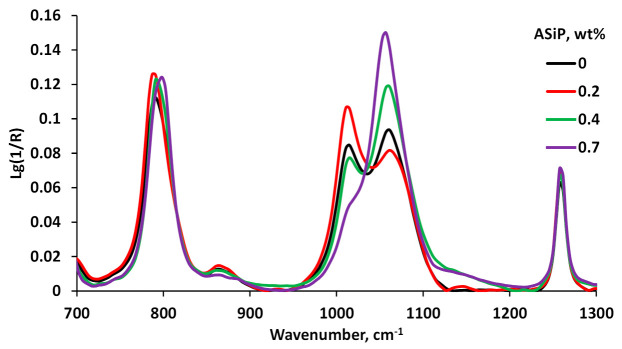
FTIR spectra of MBC based on [D_4_]:[PPEG] = 15 obtained at different ASiP contents.

The use of amphiphilic ASiP particles affects the manifestation of the FTIR spectra of the products of the interaction of PPEG with D_4_ (Figure 4). Since the band, due to stretching vibrations of the Si–C bond in the region of 795 cm^−1^, does not change its intensity, in this case, there is no reason to assert that ASiP contributes to an increase in the D_4_ conversion. For all ASiP amounts used, the analytical band at 1096 cm^−1^, due to stretching vibrations of the C–O–C bond of the polyether component, does not appear in the obtained BC, but the intensities and intensity ratios of the bands at 1018 and 1077 cm^−1^, corresponding to stretching vibrations of the Si–O–Si bond, change. With an increase in the ASiP content, a shift in the band at 783 cm^−1^, corresponding to stretching vibrations of the Si–C bond in PDMS, to 798 cm^−1^, is also observed.

Such changes are difficult to relate to the degree of conversion of D_4_. The most probable reason for such an uneven change in the position and intensities of absorption bands in the FTIR region of the spectrum can be attributed to the appearance of intermolecular interactions, of which ASiP are a participant, and their significant effect on the processes of supramolecular organization of the synthesized multi-block copolymers.

An analysis of the FTIR spectra allows us to conclude that, during the interaction of the PPEG and D_4_, the D_4_ polyaddition initiated by potassium–alcoholate groups occurs. The MBC formed in this case is the basis for further processes of microphase separation and the formation of supramolecular formations. The use of amphiphilic ASiP particles in the synthesis of MBC does not contribute to an increase in D_4_ conversion, but it affects the processes of the supramolecular organization of the obtained block copolymers.

### 3.2. Surface Active Properties of MBC

As is known, amphiphilic block copolymers consist of regions of different chemical nature and, due to this, exhibit micelle formation ability. To confirm that the formation of MBC occurs during the interaction of PPEG with D_4_ and the influence of the molar excess of D_4_ on the length of the polydimethylsiloxane component in MBC is established, their surface-active properties in the aqueous medium were studied.

In the case of the formation of MBC, hydrophobic (PDMS components) and hydrophilic (PPEG components) blocks undergo microphase separation. During micelle formation in an aqueous medium, hydrophobic blocks are associated with the formation of a core region, while the position of the hydrophilic segments will be between the core and the external aqueous medium. As a result, the hydrophobic core is stabilized by a hydrophilic shell, which serves as the interface between the bulk aqueous phase and the hydrophobic domain.

**Figure 5:**
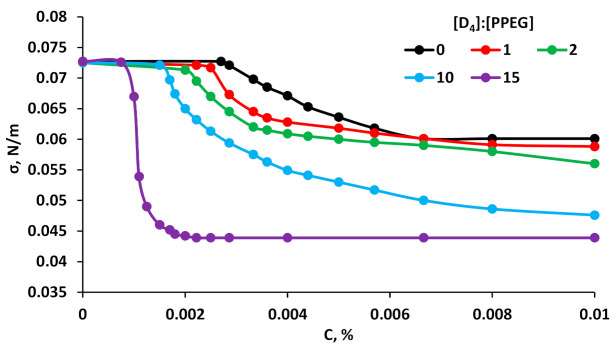
Surface tension (σ, N/m) isotherms of MBC.

According to Figure 5, noticeable changes in the concentration dependences of surface tension (σ) for MBC are observed with a twofold molar excess of D_4_ relative to PPEG. The results of measurements of the values of σ allow us to state that during the interaction of PPEG with D_4_, the D_4_ polyaddition initiated by potassium–alcoholate groups occurs with the subsequent formation of MBC. An increase in the comparative content of D_4_ leads to an increase in the size of the polydimethylsiloxane block in the composition of MBC.

**Figure 6:**
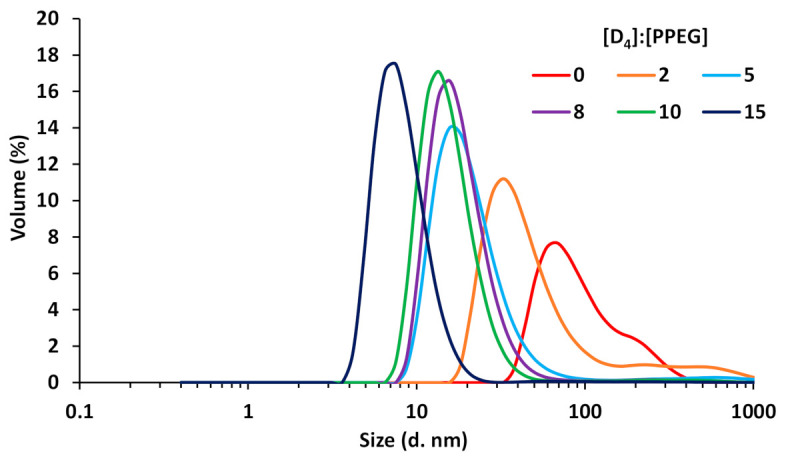
Particle size distribution in the environment of toluene.

The results of the surface tension measurements in the aqueous medium are consistent with the changes in particle size, measured in toluene (Figure 6). Thus, a decrease in particle size with an increase in the [D_4_]:[PPEG] ratio is a consequence of MBC micelle formation, the amphiphilicity of which increases with an increase in the comparative content of polydimethylsiloxane block in in their composition.

**Figure 7:**
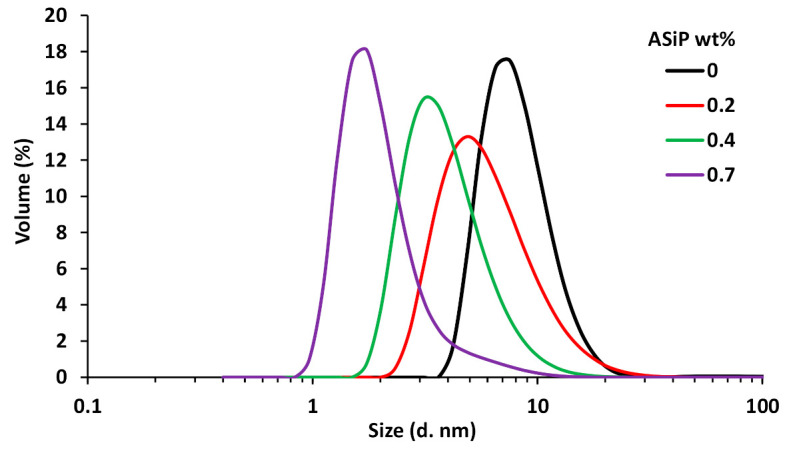
Particle size distribution in the environment of toluene.

Particle size distributions for MBC based on [D_4_]:[PPEG] = 15 with different ASiP content were also measured (Figure 7). Changing the ASiP content has a significant effect on the particle size distribution. The observed changes in the course of the particle size distribution with a change in the amount of ASiP used to modify the polymer-forming system confirm the findings of FTIR spectroscopic studies on the effect of ASiP on the macromolecular structure of MBC.

**Figure 8:**
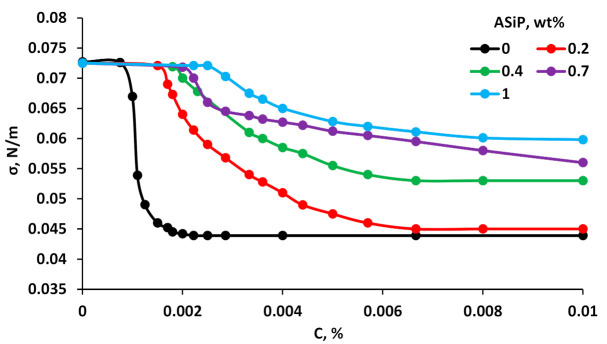
Surface tension isotherms (σ, N/m) of MBC based on [PPEG]:[D4] = 1:15 obtained at different ASiP contents.

**Scheme 2:**
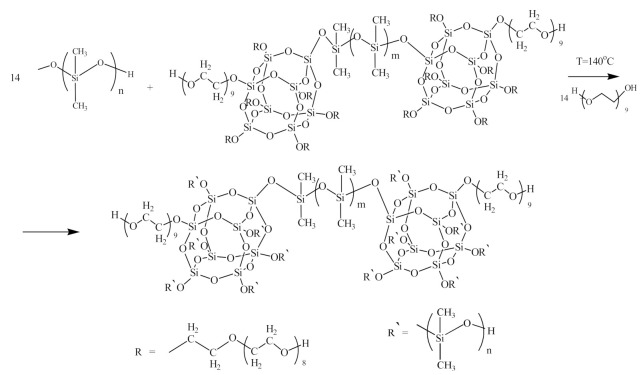
Transetherification of MBC as a result of the interaction of the terminal groups of SiOH with ASiP.

Changing the ASiP content has a significant effect on the concentration dependencies of surface tension for MBC obtained based on [PPEG]:[D_4_] = 1:15 (Figure 8). According to Figure 7 and Figure 8, with an increase in the ASiP content, the values of surface tension and critical micelle concentration noticeably increase, while the particle size decreases. This is a consequence of the fact that ASiP does not lead to an increase in the molecular weight of the PDMS block in MBC, but is the reason for its structuring. Structuring can occur due to the transetherification reaction of terminal silanol groups and ASiP (Scheme 2).

### 3.3. Polymers Characterization

**Figure 9:**
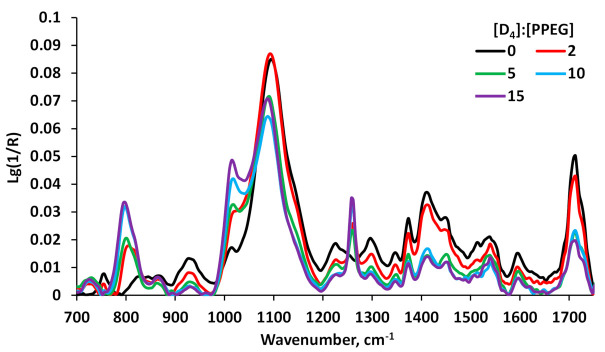
FTIR spectra of polymers based on [PPEG]:[D_4_]:[TDI] = 1:X:8.

As a next step, the polymers based on [PPEG]:[D4]:[TDI] with the different content of D_4_ were prepared. According to Figure 9, during the interaction of MBC with TDI, isocyanate groups are completely involved in the process. This is evidenced by the absence of bands in the area of 2275 cm^−1^. The formation of polyisocyanurates is evidenced by the bands in the region of 1700 and 1410 cm^−1^, due to stretching vibrations of the C=O bond present in their structure. The formation of a small number of urethane groups can be judged by the presence of a shoulder of low intensity in the region of 1730 cm^−1^, due to stretching vibrations of the corresponding C=O bonds.

**Scheme 3:**
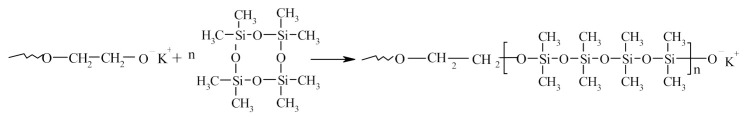
Multi-block copolymer synthesis scheme.

The results obtained allow us to describe the synthesis of amphiphilic poly(dimethylsiloxane-ethylene-propylene oxide)-polyisocyanurate cross-linked block copolymers. The formation of multi-block copolymer proceeds by the opening of octamethylcyclotetrasiloxane initiated by terminal potassium-alcoholate groups according to Scheme 3.

**Scheme 4:**
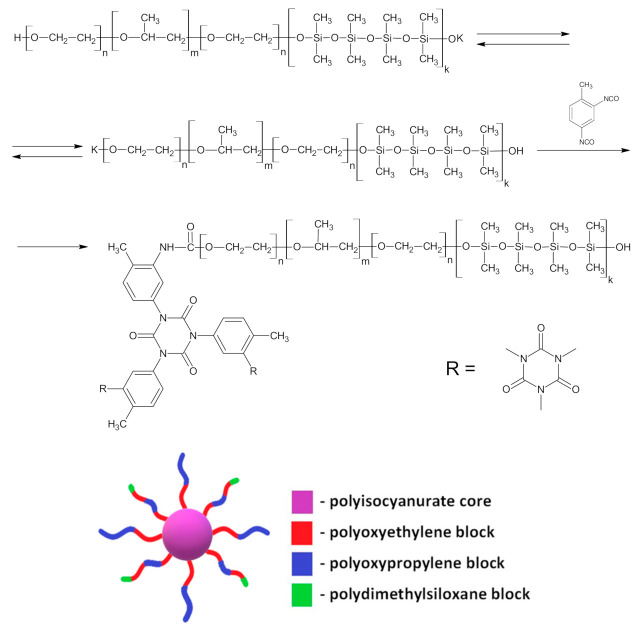
Synthesis of amphiphilic poly(dimethylsiloxane-ethylene-propylene oxide)-polyisocyanurate cross-linked block copolymers.

The migration of potassium ions in the zone of exposure to isocyanate groups of TDI creates active centers that cause the formation of isocyanurates and the formation of terminal silanol groups (Scheme 4).

As a result of a sequence of chemical reactions, isocyanurate cycles, the initiated formation of which occurs at the active centers of MBC, are combined into a single polyisocyanurate network, creating a core along the periphery of which a shell consisting of MBC is “laid”.

### 3.4. Dielectric Loss Tangents

**Figure 10:**
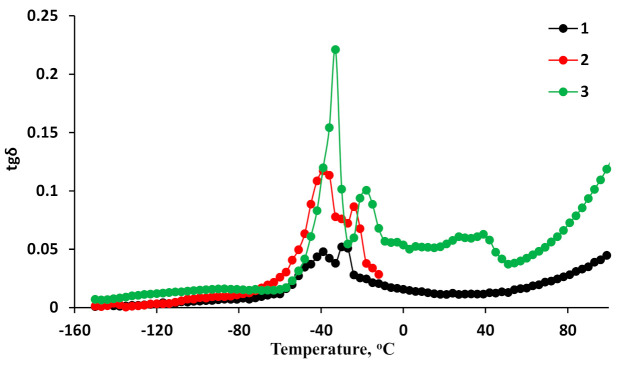
Temperature dependences of the dielectric loss tangent of polymers based on [PPEG]:[TDI] = 1:12 (1) and [PPEG]:[D_4_]:[TDI] = 1:2:12 obtained in the presence of [ASiP] = 0 wt% (2), 0.2 wt% (3).

Investigations of the temperature dependences of dielectric losses make it possible to establish the temperature of the onset of segmental mobility (*α*- and *β*-transitions) for segments merging into their own microphase, which are components of MBC (Figure 10).

In this research the reaction conditions are created where TDI enters into the PPEG-initiated TDI reaction, accompanied by the formation of rigid polyisocyanurate structures that formed the “core”. A flexible multiblock copolymer component is assembled around the “core”, creating a “shell” in the macromolecular architecture.

The polymers obtained with a small molar excess of D_4_ were investigated. According to Figure 10, for the control sample synthesized based on [PPEG]:[TDI] = 1:12 without using D_4_, one region of the α-transition is observed.

The use of even a small amount of D_4_ caused a significant change in the supramolecular organization of polymers, which was reflected in the manifestation of the temperature dependences of the *tgδ* of the polymers obtained with [PPEG]:[D_4_]:[TDI] = 1:2:12. An important consequence of the measurements is also the fact that the use of ASiP in the synthesis of the corresponding MBCs affects the microphase separation of the considered polymers.

**Figure 11:**
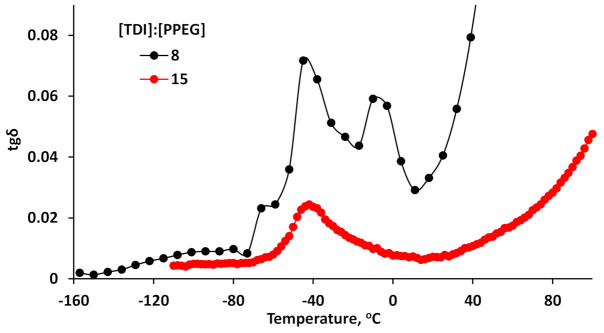
Temperature dependences of the dielectric loss tangent of polymers based on [PPEG]:[D_4_]:[TDI] = 1:15:8 and 1:15:15.

**Figure 12:**
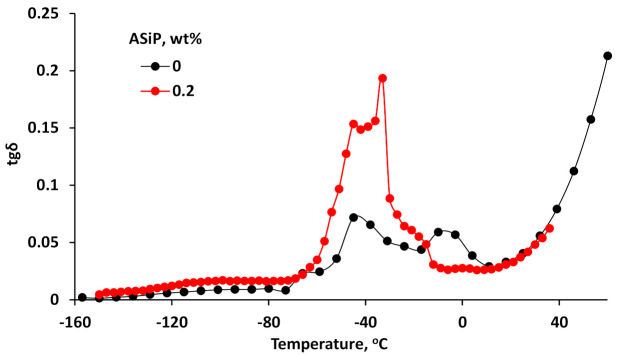
Temperature dependences of the dielectric loss tangent of polymers based on [PPEG]:[D_4_]:[TDI] = 1:15:8 with [ASiP] = 0 and 0.2 wt.%.

When the excess D_4_ is increased to the molar ratio [PPEG]:[D_4_]:[TDI] = 1:15:8, two temperature regions of *α*-transitions arise, indicating the existence of microphase separation involving polyoxyethylene (POE) and polyoxypropylene (POP) segments (Figure 11 and Figure 12). For the same ratio, a β-transition region is observed in the temperature range from −150 to −80 °C, due to the release of the polydimethylsiloxane block into its own microphase.

With an increase in the excess of TDI to the molar ratio [PPEG]:[D_4_]:[TDI] = 1:15:15, the temperature regions of the *α*-transitions are averaged, and the region of the *β*-transition is also absent in this case (Figure 11). The change in the curve of the temperature dependence of the *tgδ* for the polymer obtained on the basis of [PPEG]:[D_4_]:[TDI] = 1:15:15 can be explained by the fact that an increase in the content of TDI leads not only to an increase in the size of the “core” in the “core-shell” structure, but also an increase in the number of nodes of the spatial polymer network due to the high content of polyisocyanurates in the polymer. As a result, the implementation of a supramolecular structure built on the basis of the “core-shell” type becomes impossible.

The use of the ASiP modifier affects the processes of supramolecular organization of polymers obtained by [PPEG]:[D_4_]:[TDI] = 1:15:8. According to Figure 12, as the severity of the *β*-transition increases, the nature of the manifestation of *α*-transitions changes. The results obtained allow us to describe the principle of building the supramolecular architecture of the studied polymers as follows.

The molar excess of TDI used with respect to PPEG at a molar ratio of [PPEG]:[D_4_]:[TDI] = 1:15:8 is sufficient for crosslinking sites to be formed in the polymer matrix. Moreover, the ratio of the sizes of the polyisocyanurate “core” and the multi-block copolymer “shell” in the “core-shell” structure allows microphase separation of POE and POP segments and polydimethylsiloxane segments.

**Scheme 5:**
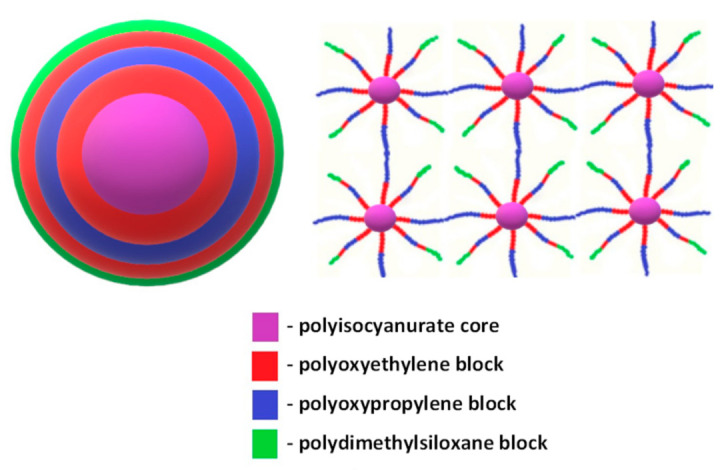
Supramolecular structure of the “core-shell” type for polymers based on MBC and TDI.

Due to the fact that the resulting polydimethylsiloxane component does not directly bind to the polyisocyanurate rigid “core”, but is located on the periphery of the supramolecular structure constructed as a “core-shell”, it extends the PPEG macrochain to an ever-greater distance from the “core”. As a result, the first layer of the “shell” consists of POE segments directly connected to the rigid polyisocyanurate core. The next is the layer of associated POP segments. The terminal POE segments, at the ends of which the initiated opening of the D_4_ cycles occurred, are pulled out from the common flexible chain PPEG due to its thermodynamic incompatibility with the polydimethylsiloxane component of the MBC chain (Scheme 5).

### 3.5. Thermomechanical Studies

**Figure 13:**
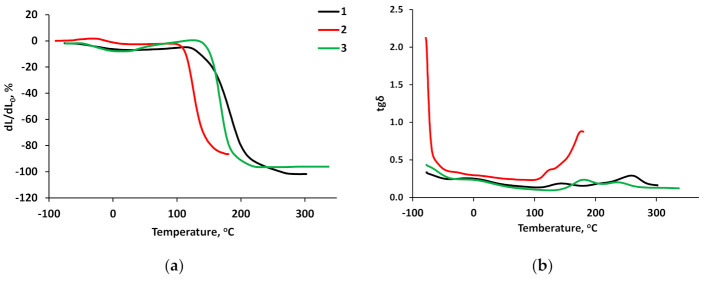
TMA curves (**a**) and temperature dependences of the mechanical loss tangent (**b**) of polymers based on [PPEG]:[TDI] = 1:8 (1) and [PPEG]:[D_4_]:[TDI] = 1:15:8 obtained in the presence of [ASiP] = 0% (2), 0.2% (3) wt.%.

According to thermomechanical studies (Figure 13), polymers obtained on the basis of [PPEG]:[TDI] = 1:8 exhibit a higher temperature onset of thermodestructive flow (T_F_) in comparison with polymers obtained on the basis of [PPEG]:[D_4_]:[TDI] = 1:15:8. The use of ASiP leads to a noticeable structuring of MBC and the polymers obtained on their basis. As a result, the T_F_ of the structured polymer increases from 114 to 153 °C.

**Figure 14:**
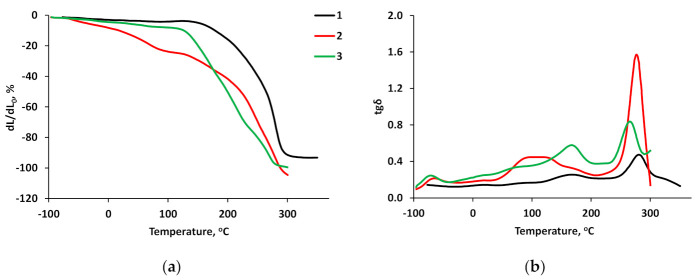
TMA curves (**a**) and temperature dependences of the mechanical loss tangent (**b**) of polymers based on [PPEG]:[TDI] = 1:12 (1) and [PPEG]:[D_4_]:[TDI] = 1:2:12 obtained in the presence of [ASiP] = 0% (2), 0.2% (3) wt%.

Apparently, the transetherification reaction involving terminal silanol groups and ASiP is the reason for the strengthening of the macromolecular and supramolecular structures of polymers obtained on the basis of [PPEG]:[D_4_]:[TDI] = 1:2:12 (Figure 14), that is, with a very low molar excess of D_4_.

**Figure 15:**
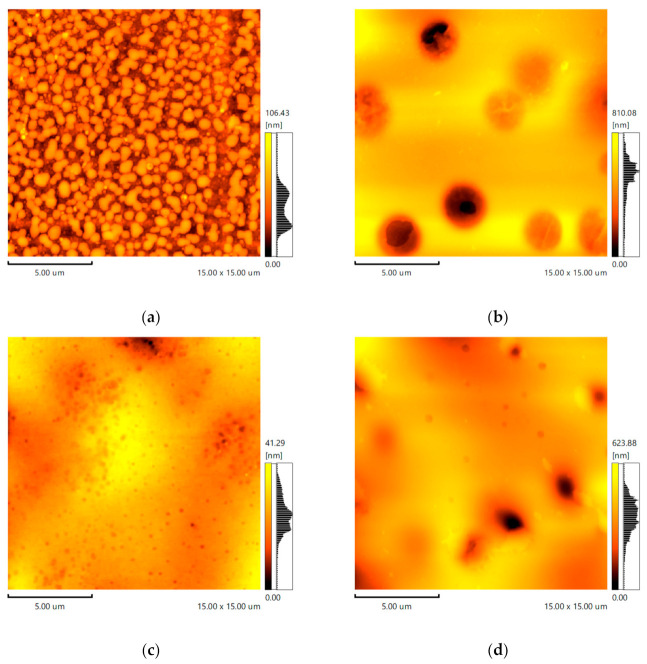
AFM Images. (**a**): [PPEG]:[TDI] = 1:10; (**b**): [PPEG]:[D_4_]:[TDI] = 1:15:10; (**c**): [PPEG]:[D_4_]:[TDI] = 1:15:10 [ASiP] = 0.2 wt.%, (**d**): [PPEG]:[D_4_]:[TDI] = 1:15:10 [ASiP] = 0.4 wt.%.

The evidence of such supramolecular organization might be also shown in the AFM images of the obtained testing examples presented on Figure 15. From the AFM images, an arithmetic average roughness height (*R*_a_) and a mean roughness depth (*R*_z_) were obtained and presented in Table 1.

### 3.6. Gas Transport Properties of Obtained Polymer Films

The resulting polymer films were investigated as gas transport membrane materials. The obtained mass transfer parameters, namely permeability, selectivity, diffusion and sorption coefficients, for the studied polymers, are given in Table 2, Table 3, Table 4 and Table 5, respectively. As the kinetic diameters of considered gases increase as following CO_2_ < N_2_ < CH_4_, the diffusion coefficient values are arranged vice versa. With regard to the sorption coefficients of studied polymers, in the case of CO_2_, these values are at least twice higher than in case of CH_4_ and almost three times higher than for N_2_. The polymers have an affinity to CO_2_, which allows for a higher sorption coefficient.

According to the work [45], the reaction conditions have a significant effect on the mechanism of TDI polyaddition initiated by the terminal potassium alcoholate groups of PPEG. Therefore, when carrying out the reaction at relatively low temperatures and in the presence of acidic cocatalysts, isocyanate groups of the para-position in the TDI are opened via the carbonyl component, followed by the formation of coplanar acetal-type polyisocyanate blocks. As a result, the supramolecular structure of such polymers acquires a cellular character. In connection with the results obtained earlier in the work [45], Table 2 shows the values of the permeability coefficient for a number of gases using a polymer with a cellular supramolecular structure obtained with [PPEG]:[TDI] = 1:15 in the presence of acidic cocatalysts as a membrane. Table 3 shows the corresponding ideal selectivity values.

Under the synthesis conditions used in this work, the opening of isocyanate groups proceeds by the usual mechanism, that is, via the N=C bond, and is accompanied by the formation of polyisocyanurates. The result of such a reaction, as shown above, is the formation of the “core-shell” type supramolecular structure. On the surface of the shell is a polydimethylsiloxane component. The use of the ASiP modifier in the synthesis leads to an additional lengthening of the polydimethylsiloxane component.

**Figure 16:**
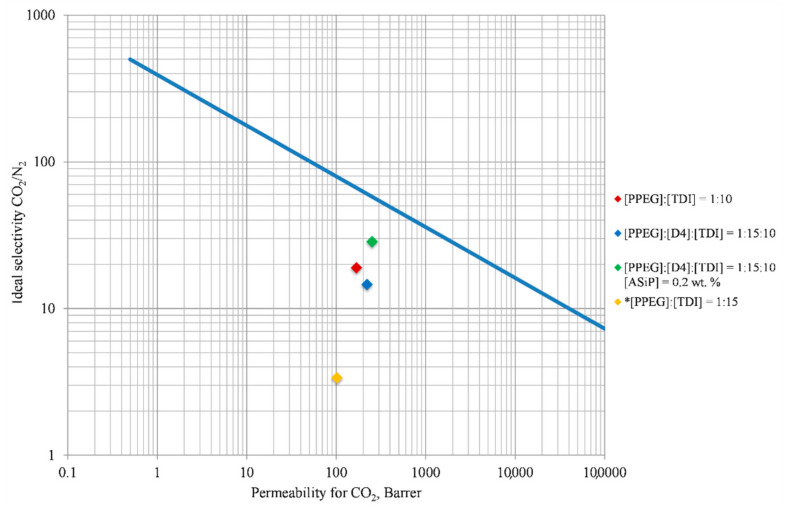
Upper bound correlation for CO_2_/N_2_ separation.

**Figure 17:**
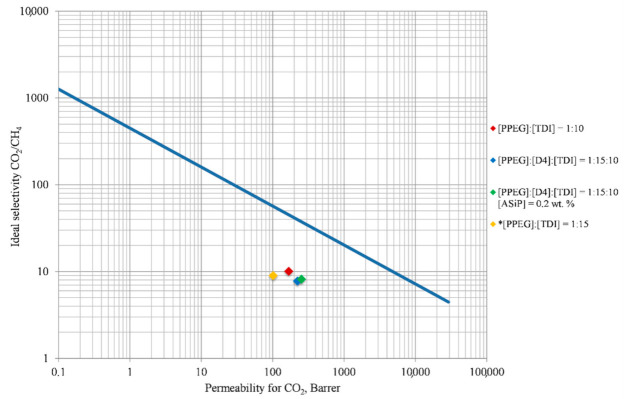
Upper bound correlation for CO_2_/CH_4_ separation.

According to the analysis of the results shown in Table 2 and Table 3, polymers with a cellular supramolecular structure exhibit lower values of carbon dioxide permeability in comparison with polymeric film materials whose supramolecular structure is constructed on the basis of the “core-shell” principle. An additional attachment of a polydimethylsiloxane component to the surface of the shell and an increase in the size of the shell lead to a sequential increase in the coefficient of permeability of carbon dioxide. In order to put results into the context of the state-of-art membrane technology, the gas transfer properties of the obtained membranes were compared to Robeson’s upper bound [53] (Figure 16 and Figure 17).

## 4. Conclusions

Multiblock copolymers obtained based on PPEG, D_4_ and TDI were investigated. It was shown that during the interaction of PPEG and D_4_, the D_4_ polyaddition initiated by potassium alcoholate groups occurs. An increase in the relative content of D_4_ leads to an increase in the polydimethylsiloxane block, which is a part of MBC. The use of the ASiP leads to the structuring of MBC via the transetherification reaction of terminal silanol groups of MBC with ASiP.

The molar ratio of PPEG, D_4_, and TDI has been established, in which the polymer chains are packed in a core-shell structure in which microphase separation of the polyoxyethylene, polyoxypropylene and polydimethylsiloxane segments, components of the shell, is carried out. Polyisocyanurates build the “core” of supramolecular formations.

The obtained polymers were studied as membrane materials for the separation of gas mixtures containing CO_2_/CH_4_ and CO_2_/N_2_. It was shown that polymers with a cellular supramolecular structure exhibit lower permeability for CO_2_ in comparison with polymeric film materials whose supramolecular structure is constructed on the basis of the “core-shell” principle. It was shown that polymers are promising as silica-based membrane materials for the separation of gas mixtures containing CO_2_/CH_4_ and CO_2_/N_2_.

## Figures and Tables

**Table 1:** Arithmetic average roughness height (Ra) and a mean roughness depth (Rz) for samples.

Sample	R_a_, nm	R_z_, nm
[PPEG]:[TDI] = 1:10	12.36	46.84
[PPEG]:[D_4_]:[TDI] = 1:15:10	90.18	402.27
[PPEG]:[D_4_]:[TDI] = 1:15:10 [ASiP] = 0.2 wt.%	4.32	19.75
[PPEG]:[D_4_]:[TDI] = 1:15:10 [ASiP] = 0.4 wt.%	6.51	32.81

**Table 2:** Permeability coefficients.

Sample	P, Barrer		
	N_2_	CH_4_	CO_2_
[PPEG]:[TDI] = 1:10	8.8	16.6	167.4
[PPEG]:[D_4_]:[TDI] = 1:15:10	9.0	28.5	221.5
[PPEG]:[D_4_]:[TDI] = 1:15:10 [ASiP] = 0.2 wt.%	8.8	30.7	251.4
[PPEG]:[TDI] = 1:15 Obtained in the presence of cocatalysts [45]	30.1	11.3	101.8

**Table 3:** Ideal selectivity for CO_2_/gas systems.

Sample	Gas System	
	CO_2_/N_2_	CO_2_/CH_4_
[PPEG]:[TDI] = 1:10	19.0	10.1
[PPEG]:[D_4_]:[TDI] = 1:15:10	14.6	7.8
[PPEG]:[D_4_]:[TDI] = 1:15:10 [ASiP] = 0.2 wt.%	28.6	8.2
[PPEG]:[TDI] = 1:15 Obtained in the presence of cocatalysts [45]	3.38	9.01

**Table 4:** Diffusion coefficients.

Sample	D × 10^10^, m^2^ s^−1^		
	N_2_	CH_4_	CO_2_
[PPEG]:[TDI] = 1:10	2.63	1.01	4.00
[PPEG]:[D_4_]:[TDI] = 1:15:10	2.70	1.20	4.10
[PPEG]:[D_4_]:[TDI] = 1:15:10 [ASiP] = 0.2 wt.%	2.64	1.28	4.40
[PPEG]:[TDI] = 1:15 Obtained in the presence of cocatalysts [45]	3.20	0.90	3.70

**Table 5:** Sorption coefficients.

Sample	S × 10^5^, mol m^−3^ Pa^−1^		
	N_2_	CH_4_	CO_2_
[PPEG]:[TDI] = 1:10	1.12	5.50	14.01
[PPEG]:[D_4_]:[TDI] = 1:15:10	1.12	7.95	18.09
[PPEG]:[D_4_]:[TDI] = 1:15:10 [ASiP] = 0.2 wt.%	1.12	8.03	19.13
[PPEG]:[TDI] = 1:15 Obtained in the presence of cocatalysts [45]	3.15	4.20	9.21

## Data Availability

The data presented in this study are available in this article.

## Abbreviations

BC	block copolymer
MBC	multiblock copolymer
PPEG	potassium-substituted block copolymer of propylene and ethylene oxide
POE	polyoxyethylene
POP	polyoxypropylene
TDI	2,4-toluene diisocyanate
D_4_	octamethylcyclotetrasiloxane
ASiP	amphiphilic branched silica derivatives associated with oligomeric medium
FTIR	Fourier transform infrared spectroscopy analysis
CMC	critical micelle concentration
TMA	thermomechanical analysis
PDMS	polydimethylsiloxane
